# Dams and tribal land loss in the United States

**DOI:** 10.1088/1748-9326/acd268

**Published:** 2023-08-09

**Authors:** Heather Randell, Andrew Curley

**Affiliations:** 1 Department of Agricultural Economics, Sociology, and Education, The Pennsylvania State University, University Park, PA 16802, United States of America; 2 Humphrey School of Public Affairs, University of Minnesota-Twin Cities, Minneapolis, MN 55455, United States of America; 3 School of Geography, Development and Environment, University of Arizona, Tucson, AZ 85721, United States of America

**Keywords:** dams, United States, Indigenous nations, infrastructure, environmental justice

## Abstract

Indigenous peoples in the United States have faced continued land dispossession for centuries. Through the reservation system as well as policies including forced removal and allotment, colonial settlers and later the federal government acquired over two billion acres from Native Nations. We argue that another important, yet understudied and unquantified, contributor to tribal land loss is through the construction of dams. By restricting water flow in rivers or lakes, dams submerge land under reservoirs and disrupt aquatic and terrestrial ecosystems. This impacts livelihoods of local communities, destroys culturally important places and resources, and displaces people from their homes and land. To quantify the amount of tribal land lost as a result of dam construction, we engage in an innovative data linkage project. We use geospatial data on the boundaries of federal Indian reservations and Oklahoma Tribal Statistical Areas (OTSAs) and overlay these data with the locations of approximately 7,900 dams in the continental US. We estimate that 139 dams have submerged over 619 000 acres of land on 56 federal reservations and that 287 dams have inundated over 511 000 acres of land on 19 OTSAs. Taken together, our lower-bound estimate is that over 1.13 million acres of tribal land have been flooded under the reservoirs of 424 dams, which amounts to an area larger than Great Smokey Mountains National Park, Grand Teton National Park, and Rocky Mountain National Park combined. In light of recent federal legislation to address aging infrastructure in the US as well as the increasing risks to dam function and safety caused by climate change, dams that impact tribal land should be prioritized for removal. In cases where removal is not a preferred or viable option, alternatives include tribal ownership or funding for repairs and improvements.

## 1. Introduction

Indigenous peoples in the United States have faced ongoing land dispossession since the arrival of European settlers more than 500 years ago. Through laws, treaties, purchases, and seizures—often advanced by violence or coercion—colonial settlers and later the federal government acquired over two billion acres from tribes [1]. Targeted policies to transfer land from tribes were implemented from the mid-1600s through the mid-20th century and included the establishment of reservations, forced removal of tribes to Indian Territory (currently Oklahoma), allotment of reservations into privately-held parcels with ‘surplus’ land sold to non-Indians, and most recently, tribal termination and relocation policies [2–5]. In this paper, we argue that an important, yet understudied and unquantified, driver of Indigenous land loss is the construction of dams.

Land dispossession is a foundation of settler colonialism, the process by which the societies, economies, and relationships to the land and water of Native Nations^4^ 4We refer to Native Nations to represent the federally recognized tribes in the United States that are the colonial experience for the hundreds of Indigenous nations that are now part of the US. We refer to both ‘Native’ and ‘Indigenous’ peoples to represent members of tribal communities impacted by damming throughout the history of federal-Indian law and policy. Indigenous peoples is a broader term and Native Nations specific to the Indigenous experience in the US. are replaced by those of settlers and their descendants [6, 7]. Land has always been central to Indigenous lifeways, and its dispossession has threatened health and well-being by displacing tribes from their traditional homelands and/or eroding the quantity and quality of land and water over which they have sovereignty [4, 8, 9]. Land loss has severed ties to family, community, culture, and ancestors; disrupted relations with the natural world; undermined food and water security; and deprived tribes of the resources with which to lead healthy lives, pursue economic activities, and generate wealth [8, 10, 11]. As Dina Gilio-Whitaker (2019: 36) [12] noted, ‘the origin of environmental injustice for Indigenous peoples is dispossession of land in all its forms’.

The colonial taking of Indigenous land has come in many forms over the past several centuries. The US initially expelled tribes from the east coast to western frontier spaces in concert with nascent imperialism and westward expansion. However, as the US consolidated most of the continent, it moved from expulsion to containment. In 1851, Congress passed the Indian Appropriations Act that set aside money to move tribes onto permanent ‘reservations.’ In the 1860s, the federal government entered treaties that established homelands for many tribes in the Great Plains and Mountain West. However, by the early 1870s, Congress banned treaty-making between tribes and the federal government, and in 1877 it passed the General Allotment Act that disregarded land rights in treaties and divided land into parcels according to tribal population under colonial surveying [13]. Through allotment alone, the federal government dispossessed tribes of more than two-thirds of the reservation land that was previously guaranteed through treaties, leaving many reservations fragmented into a patchwork of parcels owned in part by the tribe, individual tribal members, and non-Indians [10, 14]. Further, since the Supreme Court’s Lone Wolf v. Hitchcock ruling in 1903, the federal government declared the right to extinguish reservations at will, which led to the termination of several reservations in the 1950s [15, 16].

As a result of these various forms of land seizure, 42% of tribes that were historically present in the US no longer maintain any land, and among tribes that do have land today, landholdings average just 2.6% of a tribe’s original territory [17]. As of 2019, federal tribal landholdings across the entire US totaled approximately 70 million acres, which amounts to less than 3% of the country’s land area [18]^5^ 5There are multiple kinds of land tenure, all with precarious title held ‘in trust’ by the federal government. The two main types are trust and allotment lands. Trust land is the status of federally recognized reservations in the lower 48 United States whereas allotment refers to parcels of land alienated from contiguous reservation land holdings because of the General Allotment Act.. In the US, therefore, settler colonialism has resulted in the near complete taking of Indigenous land.

An underdiscussed story in the history of land dispossession, however, is the construction of dams along perennial water sources designed primarily to benefit settler communities. By impounding waterways to store, regulate, or divert water, dams typically submerge land under reservoirs. This dramatically alters the landscape and disrupts aquatic and terrestrial ecosystems [19]. Dams often flood river valleys, which have historically been densely settled due to their fertile soil and proximity to water for drinking, fishing, agriculture, and trade [20]. Dams, therefore, can have devastating impacts on communities both upstream and downstream including the loss of livelihoods, destruction of culturally important places and resources, and displacement of people from their homes and land [21–23]. Over 92 000 dams greater than six feet in height have been built in the US over the past several centuries [24]. Half are taller than 25 feet, and 2% rise more than 100 feet [25]. From the colonial era through the late 1800s, settlers constructed small and midsized dams for purposes including powering watermills, irrigating crops, and storing drinking water [26, 27]. Large, federally-owned dams proliferated in the 20th century, with the era of mega-dam construction peaking from the 1930s to 1960s [27]. Most large dams were built to serve multiple purposes simultaneously including flood control, hydropower generation, and water supply [28]. In order to fulfill these objectives, however, the dams flooded considerable amounts of land upstream and devastated social and ecological systems [19, 21].

Dams have displaced at least 40–80 million people worldwide and have negatively impacted over 472 million people downstream, including numerous Native communities in the US [21, 29]. For example, the Grand Coulee Dam on the Columbia River flooded parts of the Colville and Spokane Reservations in Washington, displacing 2,250 tribal members and decimating the salmon population, which is central to the tribes’ food security, culture, and economy [30]. Further, the Pick-Sloan Plan—a federal infrastructure project in the Missouri River basin for flood control and economic development—included a series of five large dams that flooded over 350 000 acres of land on seven reservations in North Dakota, South Dakota, and Nebraska [31]. The dams displaced numerous families and submerged farmland, forests, towns, tribal headquarters, and cemeteries [31]. Although it can be argued that many tribes have received benefits from dam building in the form of electricity, monetary compensation, or access to water for irrigation or drinking, the primary beneficiaries of dams have typically been non-Native communities [32–35]. Furthermore, the decision to build these dams was unidirectional coming out of longstanding policies of colonial control that were codified in law and practice through the doctrine of discovery and plenary power of Congress over tribes.

This paper examines the role of dams in tribal land loss. We link geospatial data on the boundaries of federal Indian reservations and Oklahoma Tribal Statistical Areas (OTSAs) to data on the location of dams and spatial extent of their reservoirs to show how additional land was lost to tribes through the construction of dams. This enables us to identify which reservations and OTSAs have lost land under reservoirs and to provide an estimate of the total acreage of tribal land submerged due to dams. Understanding the extent to which dam construction has contributed to tribal land loss is critical to broaden our knowledge of the impacts of settler colonialism and identify opportunities for reclaiming tribal sovereignty and restoring ecosystems.

## 2. Methods

In this section, we briefly describe our data and methods. Additional methodological details are included in the Methods in Detail section in the Supplementary Materials. To identify reservations and OTSAs that were affected by dams and calculate the land submerged under their reservoirs, we utilized geospatial data on the boundaries of federal reservations (including off-reservation trust lands) and OTSAs; the location of dams; and the extent of each dam’s reservoir. Data on tribal land were obtained from the US Census Bureau, which maintains a file of the current legal boundaries of American Indian, Alaska Native, and Native Hawaiian areas [36]. We focus on federal reservations and OTSAs in the continental US to cover land that was allocated to tribes by the federal government—most often by the late 1800s—prior to the construction of the majority of dams. This includes 326 reservations and 29 OTSAs.

Geospatial data on the location of dams and reservoirs were obtained from two sources: the Global Reservoir and Dam Database (GRanD) v1.3 [37, 38] and the Georeferenced global Dams And Reservoirs dataset (GeoDAR) v1.1 [39]. GRanD contains information on 1,920 large dams in the US and provides dam and reservoir names as well as numerous attributes. GeoDAR includes over 7,900 dams and reservoirs in the US (including those in GRanD), but does not provide identifying information or attributes beyond location. Both GRanD and GeoDAR provide point locations of dams and high-resolution polygons of reservoir boundaries that were obtained from remote sensing imagery [40].

To identify reservations and OTSAs on which land was submerged by dams, we overlaid geospatial data on tribal boundaries, dam locations, and reservoir extents in ArcMap version 10.8.1. Next, we calculated the amount of tribal land underlying each reservoir by intersecting the tribal boundaries with reservoir boundaries. While all dams in the GRanD database included the dam’s name, we had to manually identify each dam/reservoir that occurred only in the GeoDAR database. Due to the time-consuming nature of this process, we focused on identifying the names of all dams that flooded at least 100 acres of reservation/OTSA land. For all dams that flooded at least 100 acres of tribal land as well as those built on natural lakes, we obtained additional attribute information from the National Inventory of Dams (NID) including year of completion, owner type, owner name, primary purpose, dam height, and hazard potential [24]. See the Methods in Detail for a description of the GeoDAR dam identification process and for information on the NID data.

Because the reservoir boundaries in GRanD and GeoDAR were calculated based on satellite imagery, they capture the spatial extent of a reservoir a particular point in time—potentially during a dry period or a time of increased water demand [40]. The boundaries may therefore underestimate the extent of a reservoir when it is at capacity or when it was initially filled. For example, according to the GRanD/GeoDAR data, the Allegheny Reservoir created by the Kinzua Dam flooded approximately 3,523 acres of the Allegany Reservation, however other sources indicate that the reservoir flooded 9,000–10 000 acres of reservation land [26, 41, 42].

To more accurately reflect the full acreage of tribal land flooded under reservoirs, we replaced satellite data on area flooded with better data if available. For reservoirs located entirely within the bounds of one reservation/OTSA, we replaced satellite data with information from the NID on the reservoir’s surface area [24]. If a reservoir spanned multiple tribal boundaries and/or spanned tribal and non-tribal land, we replaced satellite data with data from the literature, if available. In addition, for three dams—the El Capitan Dam, Parker Dam, and Glen Canyon Dam—we used data from the literature because the land flooded by the dams no longer lies within contemporary reservation boundaries (see Methods in Detail for additional information on these cases). Using the updated estimates, we then quantified the total acreage of reservation and OTSA land submerged under reservoirs.

Lastly, a small portion of the dams were built on natural lakes to regulate flow or expand storage capacity, which raised lake levels and submerged land along the shorelines. In these cases, data from GRanD, GeoDAR, and NID provide the spatial extent of the entire reservoir, including that which had been part of the natural lake prior to the construction of the dam. This precludes us from identifying the spatial extent of the *additional* land area flooded when the dam was constructed. As such, we include dams built on natural lakes in our overall analysis, but exclude all but one from calculations of tribal land flooded due to data limitations.

## 3. Results

### 3.1. Federal reservation land

**Figure 1.**
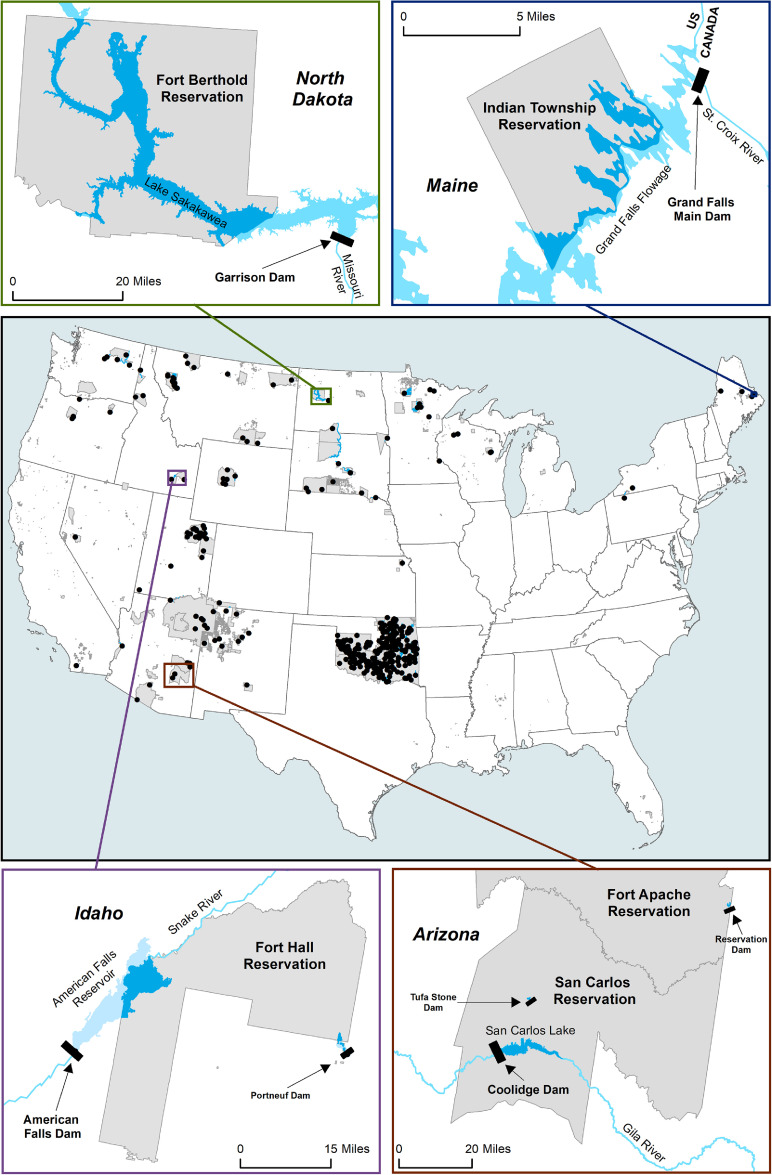
US map displaying the legal boundaries of federal reservations, off-reservation trust land, and OTSAs (in gray) and locations of dams that have flooded tribal land. Inset maps provide examples of several reservations, dams, and reservoirs, with flooded reservation land in medium blue and flooded off-reservation land in light blue.

We find that 56 of the 326 federal Indian reservations have experienced land loss due to the reservoirs of 139 dams. The vast majority of these dams were built on rivers, however 15 were constructed on natural lakes. Figure 1 shows a map of all dams that have inundated tribal land and provides several high-resolution examples. Table 1 displays summary data for the 90 dams whose reservoirs either flooded at least 100 acres of reservation land or were built on natural lakes, and table S1 in the Supplementary Materials provides detailed information on each of these dams including the affected reservation and tribe/confederation, dam and reservoir name, year completed, dam height, whether the dam is located on the reservation, main use, owner, area flooded, percent of reservation flooded by the dam, and hazard potential. An additional 49 dams flooded less than 100 acres of tribal land. For these dams, table S4 provides information on the affected reservation and tribe or confederation, the dam’s geographic coordinates, and area flooded.

**Table 1.** Summary statistics for 90 dams whose reservoirs submerged at least 100 acres of land on 42 federal reservations.

	Mean/ Proportion	Min	Max
Year completed	1947	1884	1994
Dam height (feet)	126	8	717
Reservation area flooded per dam (acres)	8,232	100	160 414
Proportion of reservation flooded	0.03	0.00001	0.32
Primary use:			
Irrigation	0.39		
Flood control	0.22		
Hydropower	0.11		
Recreation	0.11		
Water supply	0.07		
Other	0.10		
Owner type:			
Federal	0.72		
Private	0.11		
State or local government	0.08		
Tribal ownership or co-ownership	0.06		
Public Utility	0.02		
Hazard potential:			
High	0.88		
Significant	0.04		
Low	0.07		

*Notes:* Table excludes 50 smaller dams that flooded less than 100 acres of reservation land.

Calculation of reservation area flooded excludes the land flooded by 14 dams built on natural lakes as well as the Chief Joseph Dam (see Methods in Detail for more information on these cases).

For dams flooding at least 100 acres of tribal land as well as those built on natural lakes, the average year of completion was 1947, ranging from 1884 to 1994. Dams averaged 126 feet in height, however the tallest dam—the Dworshak Dam that flooded land on the Nez Perce Reservation in Idaho—rises 717 feet. On average, each flooded approximately 8,232 acres of reservation land, with the Oahe Dam flooding the most total tribal land at 160 414 acres. Dams submerged an average of 3% of a reservation’s land under their reservoirs, though the Kinzua Dam flooded nearly one-third of the Allegany Reservation. Most dams were built to serve multiple purposes simultaneously, with a primary purpose of irrigation for 39%, flood control for 22%, hydropower for 11%, recreation for 11%, and water supply for 7%. Seventy-two percent of dams are owned by the federal government, while just 6% are owned or co-owned by tribes. Finally, 88% of the dams have high hazard potential, meaning that downstream flooding due to a dam failure would likely result in the loss of human life.

We then calculated the total federal reservation land area submerged under the reservoirs of dams. We estimate that together these dams flooded at least 619 268 acres (967 square miles) of reservation land. However, it is important to note that this is a lower-bound estimate, as the true extent of tribal land flooded by dams is likely greater for several reasons.

First, our analysis is limited to dams included in GRanD and GeoDAR. Though over 92 000 dams currently impound waterbodies in the US [24], this analysis only considers the approximately 7,900 large and medium sized dams in the two databases. We are thus unable to estimate flooding associated with more than 80 000 smaller dams. For example, a report by the Bureau of Reclamation noted that the Fort Mojave, Colorado River, Fort Yuma, and Gila Bend Reservations also lost land due to federal dam projects [26], however these cases are not captured in our data.

Second, though we include dams built on natural lakes in our analysis, we exclude all but one from calculations of area flooded given that we are unable to determine the *additional* area submerged due to dam construction. The impacts of these dams, however, have been substantial [43–45]. For example, the Winnibigoshish and Leech Lake Dams, both built to impound natural lakes on the Leech Lake Reservation in Minnesota, inundated land owned by 1,300 tribal members and destroyed or damaged wild rice beds, fisheries, and forests [26]. Third, our calculations are based on reservoir surface area, which does not account for the topography of the reservoir floor and therefore underestimates land loss. Finally, our analysis does not account for additional land taken from tribes for the dam and its related infrastructure, such as roads or powerhouses. This leads to additional land loss beyond that which is submerged under a reservoir, particularly if the dam itself is located within reservation boundaries.

### 3.2. Oklahoma tribal statistical area land

Oklahoma is currently home to 39 federally-recognized tribes, however only one tribe’s land falls under federal reservation status—the Osage Reservation—with the majority of the state’s land area designated as OTSAs. OTSAs represent the boundaries of reservations from the period when the area was known as Indian Territory, prior to Oklahoma’s establishment as a state in 1907 [46]. Several tribes including the Wichita and Caddo have inhabited the area since before European colonization, and in the early- to mid-1800s, dozens of tribes from elsewhere in the US were forcibly or coercively relocated to Indian Territory from their homelands elsewhere in the US, most notably through the Indian Removal Act of 1830 [47]. Though the federal government offered land in Indian Territory to tribes in perpetuity, allotment policies and treaties enacted in the late 1800s subsequently removed the majority of the land from tribes [48]. Then when the state of Oklahoma was established, it illegally extended jurisdiction over the tribal nations in the state [49]. As a result, non-Indian settlement—as well as dam building—was more widespread on OTSA land than on reservations. For example, the majority of the city of Tulsa is located within the boundaries of the Muscogee (Creek) and Cherokee OTSAs. However, a 2020 Supreme Court decision determined that the OTSAs of five tribes covering 40% of Oklahoma—the Muscogee (Creek), Cherokee, Choctaw, Chickasaw, and Seminole Nations—remain reservations, as they were never formally disestablished by Congress [50].

**Figure 2.**
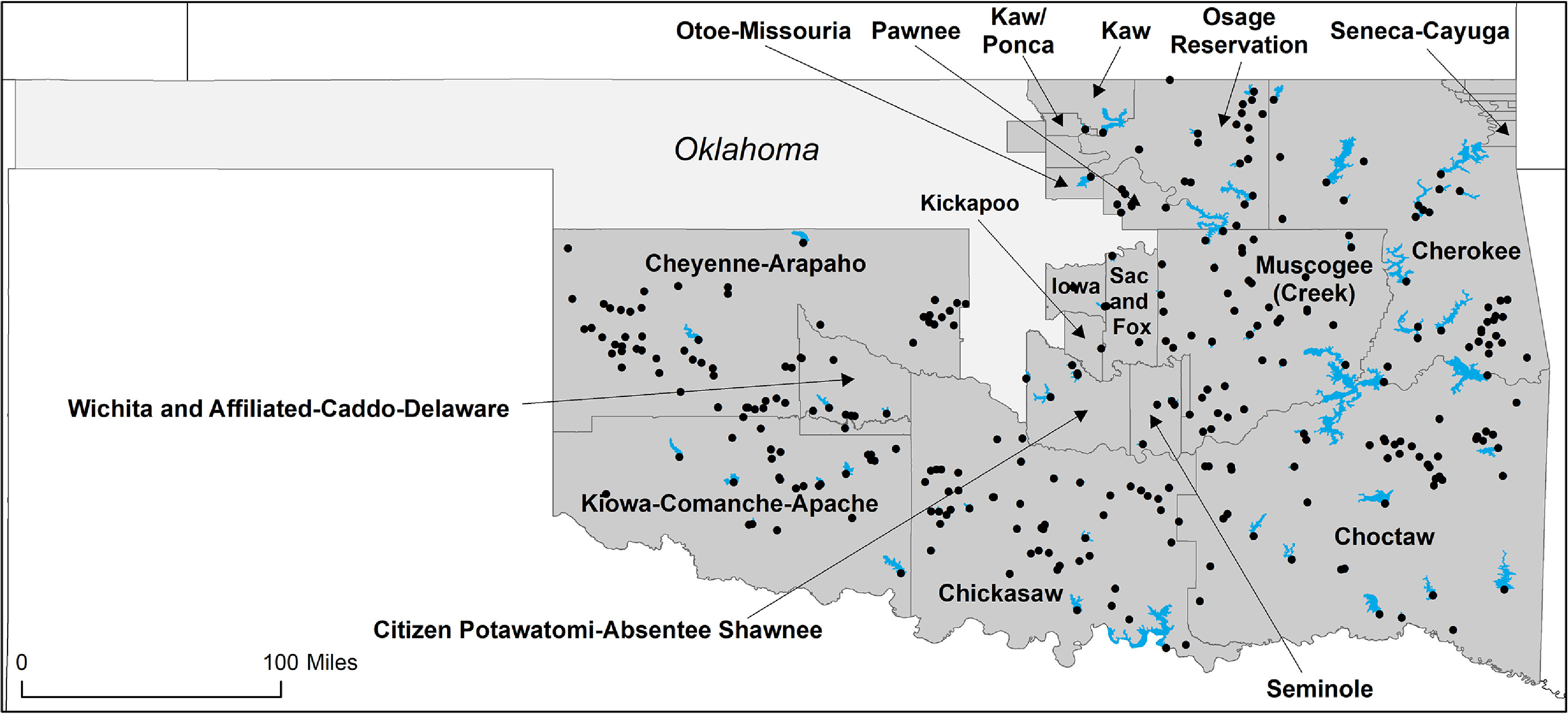
Map of Oklahoma including dams and reservoirs that affect tribal jurisdictions (OTSAs and the Osage Reservation). Tribal jurisdictions are shown in dark gray and those affected by dams are labeled.

Figure 2 shows a map of tribal jurisdictions in Oklahoma (OTSAs and the Osage Reservation) along with dams and reservoirs. According our analysis, 287 dams have flooded land on 19 OTSAs, submerging a total of 511 176 acres (799 square miles) of OTSA land. For example, the Eufaula Dam flooded land on three OTSAs: 39 607 acres of Muscogee (Creek) land, 39 531 acres of Choctaw land, and 8,575 acres of Cherokee land. Additional information on dams that flooded OTSA land is presented in the Supplementary Materials. Table S2 presents summary information on the 102 dams that flooded at least 100 acres of OSTA land and table S3 presents detailed information on each of these dams. For the 187 dams that flooded fewer than 100 acres of OTSA land, table S5 provides the name of the affected OTSA and tribe as well as the dam’s geographic coordinates and area flooded.

## 4. Discussion and conclusions

This study examined the role of dams in tribal land loss in the United States. We estimate that 139 dams have submerged over 619 000 acres of land on 56 federal reservations and that 287 dams have inundated over 511 000 acres of land on 19 OTSAs. Taken together, over 1.13 million acres of tribal land have been flooded under the reservoirs of 424 dams, which amounts to an area larger than Great Smokey Mountains National Park, Grand Teton National Park, and Rocky Mountain National Park combined. This is likely an underestimate, however, for several reasons. For example, our analysis is restricted to the approximately 7,900 large and medium dams included in the GRanD and GeoDAR databases (out of over 92 000 dams in the US). As a result of data limitations such as this, it is likely that more tribal land has been lost due to the construction of dams, with a larger number of Native Nations impacted.

Large dam construction, which peaked in the early- to mid-20th century, further eroded tribal land holdings that the US government had already substantially reduced through the establishment of reservations as well as through forced removal, allotment, tribal termination, and relocation policies [2–5, 17]. Moreover, most dams flooded river valleys—often the most fertile and densely populated land on reservations [31]—which resulted in tribes losing land that was of the greatest economic, social, and cultural value. In addition, land loss is only one of numerous ways that dams can negatively impact tribes living both upstream and downstream. For example, dams have disrupted aquatic ecosystems, decimating fisheries and wild rice beds, both of which are central to the livelihoods of many tribes [30, 51].

Tribal communities often bear the long-term costs of dams as well as other infrastructure projects. In many key infrastructure cases affecting tribal nations, tribal governments and communities tend to be the most marginalized political actors. This was true in the case of the Dakota Access Pipeline, which led to unprecedented protests in 2016 [52]. The oil pipeline now runs under Lake Oahe, threatening the drinking water supply for multiple tribes. Lake Oahe was itself created in 1962 when the Army Corps of Engineers completed the Oahe Dam on the Missouri River, flooding over 160 000 acres of land on the Standing Rock and Cheyenne River Reservations [31]. Other cases that highlight the impact of infrastructure on tribal communities are the demolition of a coal fired power plant on the Navajo Nation in 2021, and the development of water infrastructure in the State of Arizona [53]. Tribal nations are economically and politically entangled in many longstanding infrastructure projects in the US, and these projects’ economic impacts on tribes is important to consider.

Addressing aging infrastructure in the US, including dams, has been a key focus of the Biden administration. The Infrastructure Investment and Jobs Act, passed by Congress in 2021, allocates $800 million to dam removal [54]. Building on this is the 21st Century Dams Act—introduced in 2021 in the House and Senate—which would provide an additional $7.5 billion for dam removal if passed in its current form [55, 56]. Nearly 2,000 obsolete or unsafe dams have been removed in the US, however the majority were small in terms of height and/or land flooded, with only 20% of sufficient size to be included in the National Inventory of Dams [57]. In addition, climate change is undermining the function and safety of dams, as increasingly severe droughts diminish hydropower production and water supply and heavy rain events increase the risk of dam failures and downstream flooding [58, 59].

In response, dams that impact tribal land should be prioritized for federal dam removal funding given their direct role in Indigenous land loss. In assessing the viability of removing a particular dam, however, it is important to understand tribal members’ lived experiences and concerns. Indeed, the dam removal process itself can lead to stress and financial burdens for tribal members, particularly elders [60]. Dam removal efforts should therefore be implemented collectively with tribes to ensure that the interests and well-being of tribal members are reflected within the removal and restoration process. To date, there are over 30 cases of tribal involvement in dam removal within the US [61]. For example, in 2016 the Saint Regis Mohawk Tribe of New York removed the Hogansburg Hydroelectric Dam, with project land returned to the tribe and nearly 300 miles of migratory fish habitat reestablished [62]. Dam removal can act as restorative environmental justice for Indigenous communities by giving land back to tribes and rehabilitating the land and water ecosystems on which they have historically depended [61].

However, dam removal may not be viable option in all cases, for example if a dam is actively used for flood control. Further, there will be situations in which Native Nations do not favor removal, particularly cases of dams built for the benefit of tribes. In these instances, funding for repairs or improvements may be preferred. An example is the Four Horns Dam, which was built in 1931 to provide irrigation water on the Blackfeet Indian Reservation. After decades in poor condition, the dam was rebuilt in 2020 when a federal settlement provided funds for its repair [63]. In addition, the Infrastructure Investment and Jobs Act has earmarked $150 million to repair six Bureau of Indian Affairs dams over the next five years [64, 65].

Another possible response is tribal ownership, which enables tribes to manage a dam in alignment with their interests and values, and to benefit economically from jobs and revenue associated with a dam’s operation. For example, the Confederated Salish and Kootenai Tribes of the Flathead Reservation in Montana purchased the Kerr Dam in 2015, becoming the first tribes in the US to take ownership of a major hydroelectric dam [66]. The tribes renamed it the *S$\acute{e}$liš Ksanka Q$\acute{l}$isp$\acute{e}$* (Salish Kootenai) Dam, with all profits from hydropower generation accruing to the tribes and dam-related job opportunities created for tribal members.

Finally, a larger point about dams is not solely about the physical infrastructure, but also the social and political context that produced them. Dams were built on the continent’s waterways with little regard for past tribal water practices and knowledge, or for reservation land claims. As we have demonstrated throughout this paper, Indian land was not simply dispossessed from tribes in a process of settler-colonial expansion, but was also submerged in the reengineering of the continent’s rivers and lakes. At the root of these multiple forms of land and water dispossession was the disempowerment of Native people. Although dam removal is not the only solution, it is an important component that should be prioritized in federal policy. A more far-reaching resolution to the issue of existing dams and the future of waterways in the US is to amplify the power of tribes in the federal system to strengthen tribal sovereignty over their land and water.

## Data Availability

The data that support the findings of this study are openly available at the following URLs: www.globaldamwatch.org/grand/, https://zenodo.org/record/6163413#.ZCH2OOzML0r, https://nid.usace.army.mil/#/, and https://catalog.data.gov/dataset/tiger-line-shapefile-2017-nation-u-s-current-american-indian-alaska-native-native-hawaiian-area.

## Positionality Statement

We are Native and non-Native researchers in the social and environmental sciences working out of large public land-grant universities—universities with fraught histories and legacies of dispossessing Indigenous nations of land [1]. We bring our lived experiences and professional expertise on the social consequences of natural resource extraction and water infrastructure development. However, we acknowledge that Native Nations have complex relations with colonial infrastructures. We do not represent the views of tribes or individual tribal members regarding specific dams.
